# The neutralizing antibody, LY-CoV555, protects against SARS-CoV-2 infection in nonhuman primates

**DOI:** 10.1126/scitranslmed.abf1906

**Published:** 2021-04-05

**Authors:** Bryan E. Jones, Patricia L. Brown-Augsburger, Kizzmekia S. Corbett, Kathryn Westendorf, Julian Davies, Thomas P. Cujec, Christopher M. Wiethoff, Jamie L. Blackbourne, Beverly A. Heinz, Denisa Foster, Richard E. Higgs, Deepa Balasubramaniam, Lingshu Wang, Yi Zhang, Eun Sung Yang, Roza Bidshahri, Lucas Kraft, Yuri Hwang, Stefanie Žentelis, Kevin R. Jepson, Rodrigo Goya, Maia A. Smith, David W. Collins, Samuel J. Hinshaw, Sean A. Tycho, Davide Pellacani, Ping Xiang, Krithika Muthuraman, Solmaz Sobhanifar, Marissa H. Piper, Franz J. Triana, Jorg Hendle, Anna Pustilnik, Andrew C. Adams, Shawn J. Berens, Ralph S. Baric, David R. Martinez, Robert W. Cross, Thomas W. Geisbert, Viktoriya Borisevich, Olubukola Abiona, Hayley M. Belli, Maren de Vries, Adil Mohamed, Meike Dittmann, Marie I. Samanovic, Mark J. Mulligan, Jory A. Goldsmith, Ching-Lin Hsieh, Nicole V. Johnson, Daniel Wrapp, Jason S. McLellan, Bryan C. Barnhart, Barney S. Graham, John R. Mascola, Carl L. Hansen, Ester Falconer

**Affiliations:** 1 Lilly Biotechnology Center, Eli Lilly and Company, San Diego, CA 92121, USA.; 2 Eli Lilly and Company, Indianapolis, IN 46225, USA.; 3 Vaccine Research Center, National Institute of Allergy and Infectious Diseases, National Institutes of Health, Bethesda, MD 20892, USA.; 4 AbCellera Biologics Inc., Vancouver, BC V5Y0A1, Canada.; 5 University of North Carolina at Chapel Hill, Chapel Hill, NC 27599, USA.; 6 Galveston National Laboratory and Department of Microbiology and Immunology, University of Texas Medical Branch, Galveston, TX 77555, USA.; 7 Department of Population Health, Division of Biostatistics, New York University Grossman School of Medicine, New York, NY 10016, USA.; 8 Department of Microbiology, New York University Grossman School of Medicine, New York, NY 10016, USA.; 9 NYU Langone Vaccine Center, Department of Medicine, Division of Infectious Diseases and Immunology, New York University Grossman School of Medicine, New York, NY 10016, USA.; 10 Department of Molecular Biosciences, University of Texas at Austin, Austin, TX 78712, USA.

## Abstract

Severe acute respiratory syndrome coronavirus-2 (SARS-CoV-2) poses a public health threat for which preventive and therapeutic agents are urgently needed. Neutralizing antibodies are a key class of therapeutics that may bridge widespread vaccination campaigns and offer a treatment solution in populations less responsive to vaccination. Here, we report that high-throughput microfluidic screening of antigen-specific B cells led to the identification of LY-CoV555 (also known as bamlanivimab), a potent anti-spike neutralizing antibody from a hospitalized, convalescent patient with coronavirus disease 2019 (COVID-19). Biochemical, structural, and functional characterization of LY-CoV555 revealed high-affinity binding to the receptor-binding domain, angiotensin-converting enzyme 2 binding inhibition, and potent neutralizing activity. A pharmacokinetic study of LY-CoV555 conducted in cynomolgus monkeys demonstrated a mean half-life of 13 days and a clearance of 0.22 ml hour^−1^ kg^−1^, consistent with a typical human therapeutic antibody. In a rhesus macaque challenge model, prophylactic doses as low as 2.5 mg/kg reduced viral replication in the upper and lower respiratory tract in samples collected through study day 6 after viral inoculation. This antibody has entered clinical testing and is being evaluated across a spectrum of COVID-19 indications, including prevention and treatment.

## A monoclonal for SARS-CoV-2

Among the most promising therapeutic options for individuals with coronavirus disease 2019 (COVID-19) are monoclonal antibodies (mAbs). In this study, Jones *et al*. identified, characterized, and tested one such mAb, LY-CoV555, in vitro and in vivo. They found that LY-CoV555 bound to the severe acute respiratory distress syndrome coronavirus-2 (SARS-CoV-2) spike protein and prevented its interaction with angiotensin-converting enzyme 2. Prophylactic treatment with LY-CoV555 protected the upper and lower respiratory tracts of nonhuman primates from becoming infected with SARS-CoV-2. Together, these data support the clinical use of LY-CoV555 for treating patients with COVID-19.

## Teaser

LY-CoV555, a SARS-CoV-2 spike protein–specific antibody, neutralizes SARS-CoV-2 and protects the airways of nonhuman primates against infection.

## INTRODUCTION

The global coronavirus disease 2019 (COVID-19) pandemic continues to spread rapidly with substantial health, economic, and societal impact (*1*). Severe acute respiratory syndrome coronavirus-2 (SARS-CoV-2), the coronavirus responsible for COVID-19, can induce acute respiratory distress syndrome and a wide spectrum of symptoms leading to substantial morbidity and mortality (*2*). Neutralizing antibodies represent an important class of therapeutics that could provide immediate benefit in treatment or as passive prophylaxis until vaccines are widely available. Passive prophylaxis could be an alternative to vaccination in populations where vaccines have been found to be less efficacious (*3*, *4*). The capabilities required to rapidly identify, test, and ultimately manufacture antibodies have been established (*5*–*7*), which provide a path to make the most of individuals who have been infected in the early stages of a pandemic as a source of neutralizing antibodies that could be deployed rapidly for prevention and treatment of viral infection.

SARS-CoV-2 neutralizing antibody discovery efforts, including this study, have focused on targeting the multidomain surface spike protein, a trimeric class I fusion protein that mediates viral entry. Spike protein–dependent viral entry is initiated by upward movement of the receptor-binding domain (RBD) at the apex of the protein allowing access to bind the angiotensin-converting enzyme 2 (ACE2) cellular receptor (*8*–*11*). Upon receptor engagement, coordinated proteolytic cleavage, shedding of the S1 subunit, and conformational rearrangement of the S2 subunit lead to viral fusion with the cell and transfer of genetic material. Given the critical nature of the RBD interaction with ACE2 for viral entry, antibodies that bind the RBD and interfere with ACE2 binding can have potent neutralizing activity (*7*, *12*, *13*), some of which have progressed to clinical study (*14*).

To test the potential for neutralizing monoclonal antibodies (mAbs) to prevent SARS-CoV-2 infection in vivo, we used the rhesus macaque challenge model. Although rhesus macaques do not exhibit the severe pulmonary symptoms sometimes associated with human COVID-19 disease, the model allows for assessment of viral replication in the upper and lower airways (*15*–*19*). Of particular interest, recent studies in this model have shown that prior exposure to SARS-CoV-2 or administration of a SARS-CoV-2 vaccine is sufficient to prevent infection upon subsequent challenge (*18*, *20*). Protecting nonhuman primates (NHPs) from SARS-CoV-2 infection may inform the clinical development of medical countermeasures for patients with COVID-19 (*17*, *21*).

In this study, we report a strategy for high-throughput screening, which allowed for the rapid identification and subsequent characterization of anti-spike neutralizing antibodies. An RBD-specific antibody (LY-CoV555) was found that binds to the RBD in the up (active) or down (resting) conformation and demonstrated substantially greater neutralization potency of SARS-CoV-2 in vitro relative to all other antibodies analyzed from this patient. Passive immunization by infusion of LY-CoV555 protected both lower and upper airways from SARS-CoV-2 infection in a rhesus macaque model. These data supported the rapid progression of LY-CoV555 into clinical evaluation, where single antibody efficacy in the treatment of SARS-CoV-2 infection was subsequently demonstrated (*22*).

## RESULTS

### Identification of convalescent patient–derived SARS-CoV-2 antibodies

**Fig. 1:**
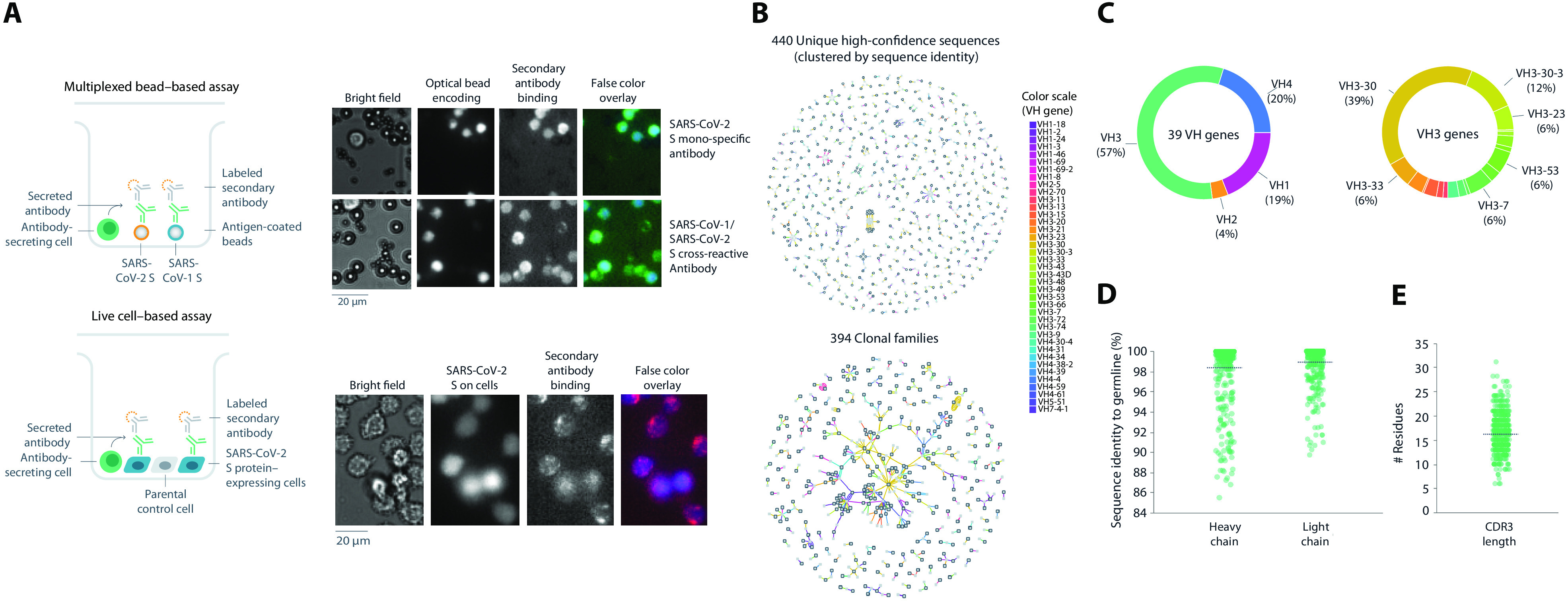
Antibody screening and sequence analysis. (**A**) Representation of multiplexed bead–based and live cell–based screening assays. Representative microscopic images of antibodies assessed for SARS-CoV-2 spike protein specificity in each indicated assay. (**B**) Sequence analysis of the 440 unique high-confidence paired-chain antibodies. Graphical representation of antibodies clustered according to sequence identity (top) or clonal family relationships (bottom). Each node indicates a chain or a cluster of chains. Heavy chains are outlined in black. Each line connecting the nodes indicates a single antibody, colored by VH gene usage according to legend. Multiple lines that connect to the same heavy- and light-chain clusters represent clonally related antibodies. (**C** to **E**) Sequence profiles of antibodies showing VH gene usage (C), distributions of sequence identity to germline for heavy and light chains (D), and CDR3 length (E). CDR3, complementary-determining region 3; VH, heavy chain variable.

To identify potential therapeutic antibodies from a convalescent patient after diagnosis with COVID-19, a high-throughput screening approach was used to identify relevant anti-spike mAbs (Fig. 1). Peripheral blood mononuclear cells (PBMCs) were obtained about 20 days after symptom onset. Two screening assays were used: (i) a multiplexed bead–based assay using optically encoded microbeads, each conjugated to either soluble prefusion-stabilized trimeric SARS-CoV-2 or SARS-CoV-1 spike protein, and (ii) a live cell–based assay using mammalian cells that transiently expressed full-length membrane-anchored SARS-CoV-2 spike protein (Fig. 1A). In total, 5.8 million PBMCs were screened and machine learning (ML)–based analysis pipelines were used to automatically select and rank >4500 antibody “hits” (0.08% frequency), of which 2238 single antibody-secreting cells were chosen for recovery. Next-generation sequencing (NGS) libraries of antibody genes from selected single B cells were generated and sequenced, and a custom bioinformatics pipeline with ML-based sequence curation was used to recover paired-chain antibody sequences, resulting in 440 unique high-confidence paired heavy- and light-chain sequences (Fig. 1B). The sequences belonged to 394 clonal families and used a diverse set of 39 heavy-chain variable (VH) genes, with the VH3 family of genes representing 57% of total diversity (Fig. 1C), similar to other reports (*23*). Among these, the VH3-30 gene was the most common (39%). Of the 440 unique antibodies identified, 4% were cross-reactive to both full-length SARS-CoV-2 and SARS-CoV-1 spike proteins. The mean sequence identity to germline was high (98 and 99% for heavy and light chains, respectively) (Fig. 1D) with a broad distribution of complementarity-determining region 3 (CDR3) lengths (Fig. 1E), likely due to sample collection early in the immune response.

### Down-selection and binding characterization of SARS-CoV-2 antibodies

**Fig. 2:**
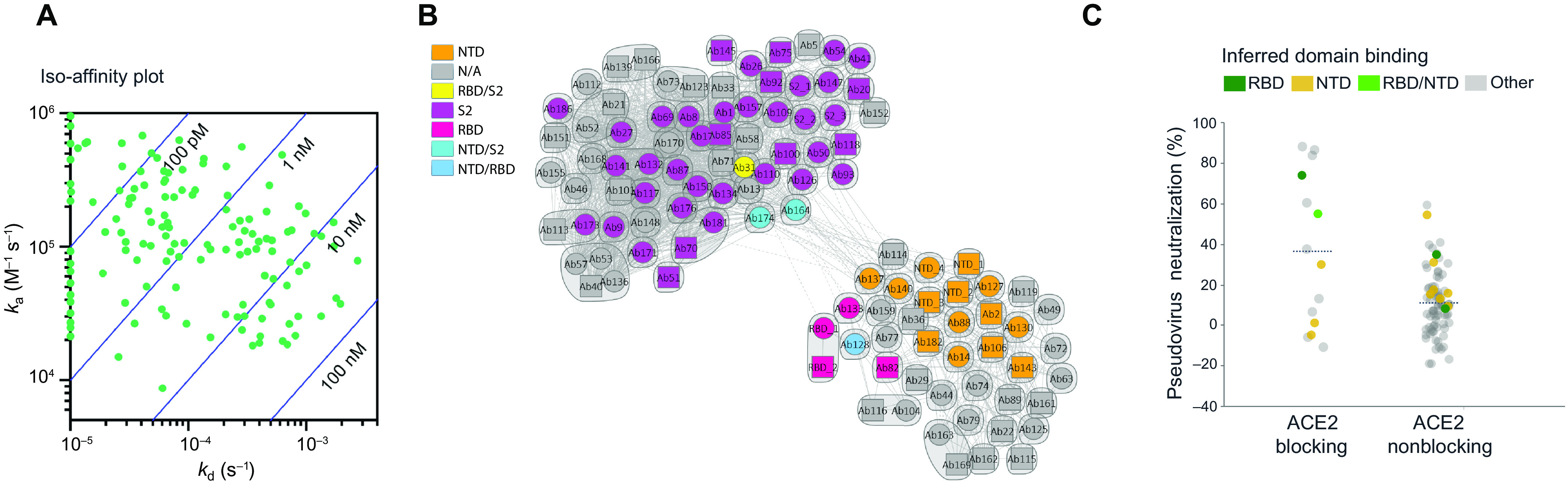
Binding validation, kinetic analysis, and pseudovirus neutralization of SARS-CoV-2 spike protein–specific antibodies. (**A**) Iso-affinity plot showing binding kinetics of recombinantly expressed antibodies. Association and dissociation rate constants were measured by high-throughput surface plasmon resonance (SPR) capture kinetic experiments with antibodies as immobilized ligands and antigens of interest as analytes. The distribution of kinetic values is displayed in an iso-affinity plot. Blue diagonal lines represent *K*_d_ values. *k*_a_, association kinetic rate constant; *k*_d_, dissociation kinetic rate constant; *K*_d_, binding affinity constant. (**B**) Competition plot of recombinantly expressed antibodies. Each antibody was tested in two orientations: as a ligand on a chip and as an analyte in solution. Individual antibodies are represented either as a circle (data present in both orientations) or as a square (data present with the antibody in a single orientation). Bins are represented as envelopes (95 total) and competition between antibodies as solid (symmetric competition) or dashed (asymmetric competition) lines. Benchmark-based blocking profiles are indicated by color. N/A, not available; RBD, receptor-binding domain; NTD, N-terminal domain. (**C**) Pseudovirus neutralization activity relative to ACE2-blocking profile of antibodies. Where available, inferred binding domains of antibodies to RBD and NTD are indicated by color.

From the set of 440 antibodies, we used an internally developed informatics and data visualization software package, Celium, to select 187 antibodies for rapid cloning and recombinant expression. Preference was given to antibodies observed at high frequency across the dataset, especially those found in both multiplexed soluble protein and live-cell assays. The selection also maximized the diversity of VH genes and CDR3 sequences and limited CDR3 sequence liabilities. A total of 175 sequences were successfully cloned into expression vectors to generate recombinant antibodies with immunoglobulin G1 (IgG1) backbones for more detailed characterization. Subsequent characterization included high-throughput biophysical analysis (fig. S1A), validation of soluble and cell-associated spike protein binding, cross-reactivity to other coronavirus spike proteins and three circulating SARS-CoV-2 spike variants (fig. S1B), apparent binding affinity to soluble spike by surface plasmon resonance (SPR) (Fig. 2A and fig. S1C), and functional screening in a high-throughput pseudotyped lentivirus reporter neutralization assay.

Of the 175 selected antibodies, 92% of antibodies were validated as SARS-CoV-2 binders, 34% as bat SARS-like coronavirus WIV1 binders, 31% as SARS-CoV-1 binders, 3% as human coronavirus HKU1, 2% as Middle Eastern respiratory syndrome coronavirus (MERS-CoV) binders, and 2% as cross-binders to all spike proteins (fig. S1B). Furthermore, 51% of antibodies were validated as SARS-CoV-2 S1 subunit–specific binders, with 8% cross-binding to full-length WIV1 and 6% cross-binding to full-length SARS-CoV-1, suggesting that, as expected, most cross-binders are S2 subunit–specific. Antibody binding to cell-expressed, full-length SARS-CoV-2 wild-type spike and known circulating variants (V367F, V483A, and D614G) was validated via automated high-throughput flow cytometry (fig. S1B). In this assay format, 77% of antibodies were validated as wild-type spike protein binders. Of that subgroup, 93% were also validated for binding to two RBD mutations (V378F and V483A) and the very common D614G non-RBD mutation. In addition, 76% of antibodies were validated in both multiplexed bead–based and live cell–based assays (fig. S1B), indicating the robustness of the single-cell screening assays with integrated ML-based hit detection for identifying SARS-CoV-2–specific antibodies. Consistent with the bead- and cell-based binding studies, these antibodies exhibited high-affinity binding to the soluble spike protein in SPR capture kinetic experiments using a Carterra LSA instrument (Fig. 2A and fig. S1C). Of these, 53% of the selected antibodies had apparent binding affinity constant (*K*_d_) values in the picomolar range and the remaining 47% had apparent *K*_d_ values in the nanomolar range, with a mean *K*_d_ value of 5.3 nM. Because of the trimeric nature of the soluble spike protein and the potential bivalent binding by the coupled antibodies, these affinities are substantially greater than true monomeric binding affinities (table S1), but likely are more representative of the pharmacological setting.

High-throughput SPR experiments were used to characterize the epitope coverage of the 175 antibodies. These experiments included antibody pairing, isolated domain binding, and binding competition with ACE2 (Fig. 2). Benchmark antibodies with known binding to the S1 subunit, N-terminal domain (NTD), RBD, and S2 subunit epitopes of the SARS-CoV spike protein and cross-reactivity to SARS-CoV-2 spike protein were included to mark epitope identity. Antibody cross-blocking results are summarized in a competition plot (Fig. 2B), as well as in a heatmap (fig. S2). In total, 95 unique bins (including controls) were identified, and a clear divide between S1- and S2-specific antibodies, as inferred by benchmark competition, was observed (fig. S2), suggesting that these antibodies had a broad epitope diversity. Only about 10% of the antibodies tested exhibited ACE2 competition. Antibodies with ACE2 binding inhibition properties had the greatest neutralizing activity based on pseudotyped lentivirus reporter neutralization (Fig. 2C), although antibodies to other domains also had detectable neutralizing activity.

A lead panel of 24 antibodies (table S2) was selected using the Celium software, on the basis of the following criteria: (i) binding to SARS-CoV-2 spike protein in either the multiplexed bead–based or the live cell–based validation assay, (ii) >30% pseudovirus neutralizing activity at any of the concentrations tested (10, 1, 0.1, or 0.01 μg/ml), (iii) dose-dependent neutralization profile, (iv) RBD competition, (v) ACE2 blocking activity, and (vi) acceptable biophysical profile (melting temperature, solubility, and polydispersity). The selected antibodies were then produced at a larger scale for further testing. The binding properties of these selected antibodies, specifically binding to which domain of the spike protein, apparent antibody affinity to the trimeric spike protein, and monomeric Fab binding affinities are summarized in table S1; despite a relatively narrow range of fully avid antibody binding to the spike protein with nearly all apparent affinities falling within a 100-fold window, monomeric Fab binding was much more variable and substantially weaker (fig. S3). As expected from the diverse nature of these properties, these antibodies exhibit a range of competition behavior with each other, leading to a number of epitope communities (fig. S4).

### Binding epitope characterization of SARS-CoV-2 antibodies

Using negative-stain electron microscopy (nsEM), we were able to further structurally characterize the binding of a subset of these antibodies (fig. S5A). Images of sufficient quality to enable three-dimensional (3D) reconstructions of Fab-spike protein complexes for five of the Fabs were collected for three RBD binders (Ab104, Ab138, and Ab169) and two NTD binders (Ab89 and Ab130). Although the individual antibodies have unique epitopes exhibiting different orientations of the Fab relative to the spike protein, similarities and overlaps were observed between them (fig. S5A). We also used hydrogen-deuterium exchange (HDX) followed by mass spectrometry (MS; table S3) to obtain epitope information for antibodies not observable by nsEM and to gain finer epitope sequence detail for several antibodies. Consistent with nsEM experiments for antibodies characterized by both methods, peptides exhibiting protection from exchange resided within the expected structural regions. Epitope information was also obtained for an additional five RBD binders, three NTD binders, and three antibodies where protection from HDX was not localized to a single domain (Ab82) or S2 binders (Ab127 and Ab164).

### Neutralization activities of SARS-CoV-2 antibodies

**Fig. 3:**
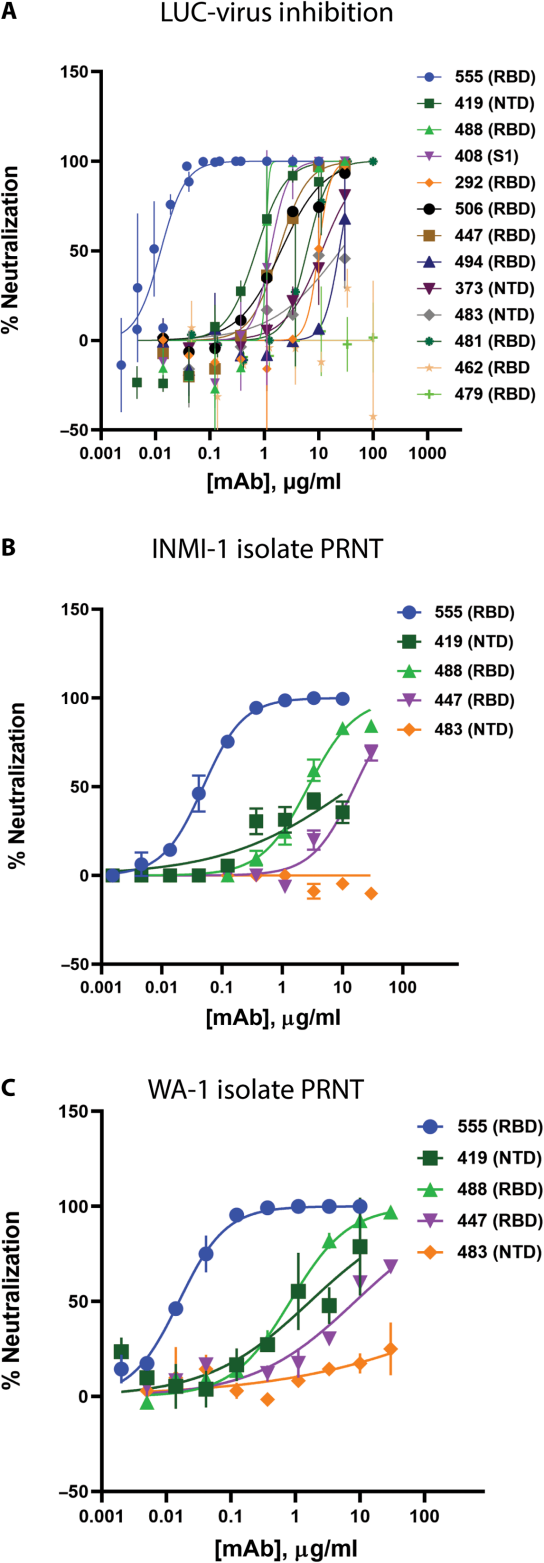
In vitro neutralization of SARS-CoV-2. (**A**) Neutralization of recombinant SARS-CoV-2 encoding a nanoluciferase reporter in the Orf7a/b locus (Luc-Virus) in infected Vero E6 cells 24 hours after inoculation is shown. Values plotted are means of two replicates (*n* = 2), with error bars showing SEM. (**B** and **C**) Results from plaque reduction neutralization test (PRNT) assays for INMI-1 isolate (B) and 2020/USA/WA-1 isolate (C) of SARS-CoV-2 in Vero E6 cells 72 hours after inoculation are shown. Values plotted are means of two replicates (*n* = 2), with error bars showing SEM. mAb, monoclonal antibody; RBD, receptor-binding domain; NTD, N-terminal domain.

The selected antibodies had a broad range of neutralizing activity in multiple in vitro assays, including pseudovirus (table S4) and various live virus assay formats. Using a replication-competent SARS-CoV-2 molecular clone in which a nonessential gene (ORRF7) has been replaced with a nano-luciferase reporter (Fig. 3A), neutralizing activity values spanning nearly three orders of magnitude were observed (table S4). For a smaller number of antibodies, viral neutralization was further characterized in a plaque reduction neutralization test (PRNT) format against two different clinical SARS-CoV-2 isolates, the INMI-1 isolate (clade 19A, Fig. 3B) and the USA/WA-1/2020 isolate (clade 19B, Fig. 3C), representing two major clades of SARS-CoV-2 (www.gisaid.org). The spike protein sequences for both isolates are identical to the Wuhan-Hu-1 isolate sequence (National Center for Biotechnology Information reference sequence entry NC_045512.2). It was observed that some non–RBD-binding antibodies, for example, Ab82, Ab89, and Ab130, exhibited greater neutralizing activity in some of the live virus SARS-CoV-2 assays compared to pseudovirus assays (table S4). The neutralization potency of one mAb, Ab169 (designated as LY-CoV555), an RBD binder and ACE2 blocker, was consistently and substantially greater than the rest and was selected for further development.

**Fig. 4:**
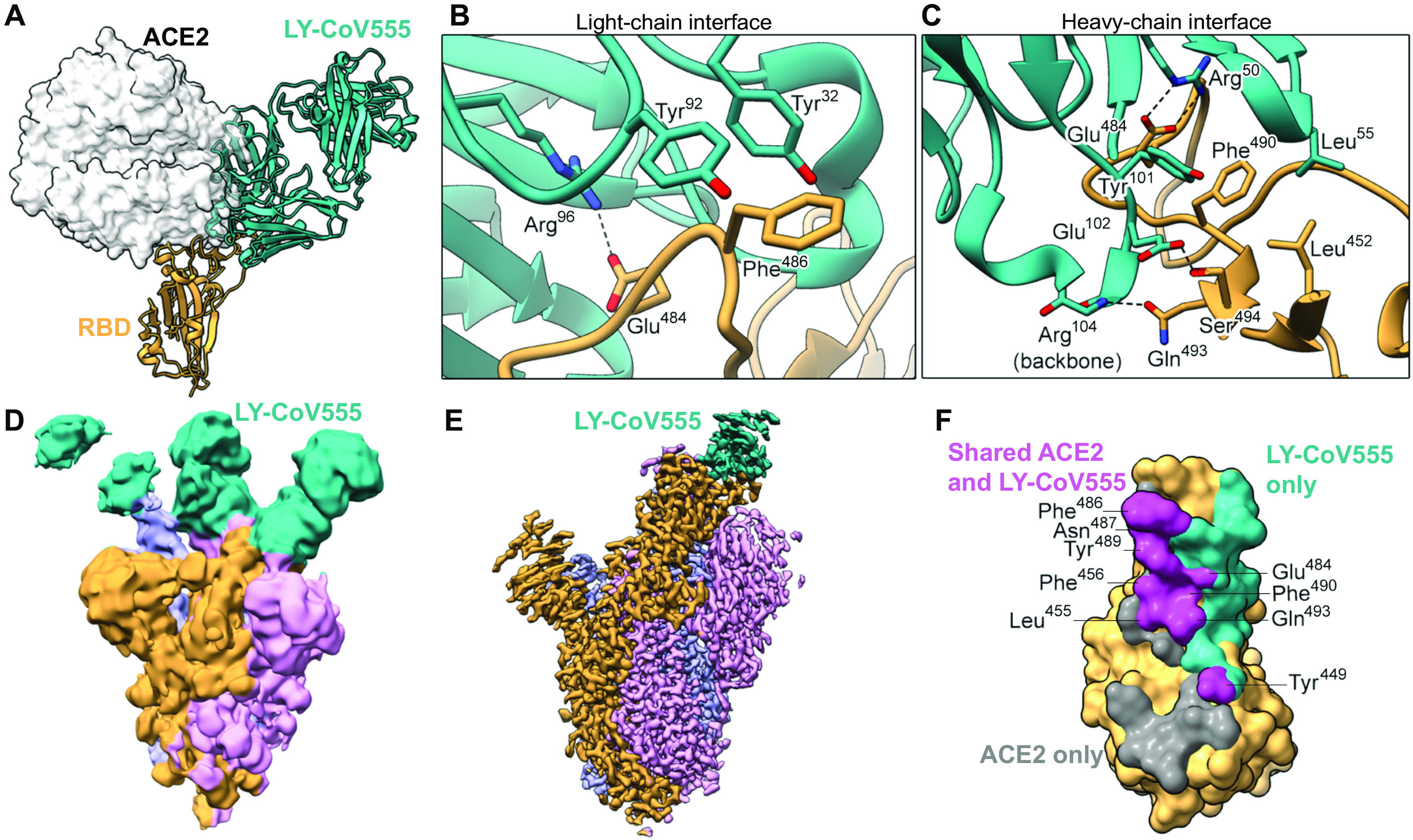
LY-CoV555 blocks ACE2 and binds to the spike protein RBD in up and down conformations. (**A**) Crystal structure of the RBD-LY-CoV555 complex superimposed with the ACE2 receptor from a structure of the RBD-ACE2 complex (Protein Data Bank ID: 6M0J) (*83*). (**B** and **C**) Zoomed-in view of key atomic interactions at the interface of the LY-CoV555 light chain (B) and heavy chain (C) with the spike RBD. (**D**) Cryo-EM structure of the LY-CoV555 spike complex low-pass–filtered to 8-Å resolution and shown at low threshold to visualize all three Fabs (shown in cyan). (**E**) High-resolution cryo-EM map of the LY-CoV555-spike complex. (**F**) SARS-CoV-2 RBD molecular surface, with the portion of the surface that only interacts with ACE2 (gray), only interacts with LY-CoV555 (cyan), or interacts with both ACE2 and LY-CoV555 (pink). Interacting atoms were defined as being within 5.5 Å of each other, and the residues containing atoms interacting with both ACE2 and LY-CoV555 are labeled. Cryo-EM, cryo–electron microscopy; RBD, receptor-binding domain.

LY-CoV555 had substantially (>10-fold) greater neutralization potency relative to other identified RBD-binding and ACE2-blocking antibodies, such as Ab128 and Ab133, despite having similar apparent binding affinities (table S2), suggesting a distinct binding mode of recognition. Structural analysis using x-ray crystallography and cryo–electron microscopy (cryo-EM) demonstrated that two of the RBD-binding mAbs (Ab128 and Ab133) bind in a nearly identical fashion to one another (fig. S5B), differing from LY-CoV555 and yet nearly identical in site and orientation to the previously described mAb CB6 (also known as etesevimab) (*13*). The epitope recognized by Ab128, Ab133, and CB6 only becomes exposed on the RBD after its transition from the down to the up and active state of the RBD. LY-CoV555 was observed to bind to an epitope overlapping the ACE2 binding site (Fig. 4, A to C); specifically, 7 of the approximate 25 side chains in the RBD were observed to form contact with ACE2 (*8*, *24*, *25*). Structural information was used to map the portion of the RBD molecular surface that interacts with ACE2 only, LY-CoV555 only, or both (Fig. 4D). On the basis of the crystal structure, the LY-CoV555 epitope, which has some overlap with the epitope for Ab128, Ab133, and CB6 described above, was predicted to be fully accessible on both the up and down conformations of the RBD. This was confirmed by high-resolution cryo-EM imaging of LY-CoV555 Fab complexes in which the LY-CoV555 Fab was observed to bind the spike protein RBD in both up and down conformations (Fig. 4, E and F, and fig. S6). This property is reminiscent of the binding of the Ebola virus–specific mAb114 that binds the Ebola virus glycoprotein RBD in both the preactivation and activated states (*26*). mAb114 was subsequently shown to effectively treat Ebola disease as monotherapy (*27*), suggesting an advantage for mAbs that can bind critical functional domains of class I fusion proteins at multiple stages of the entry process.

### LY-CoV555 provides protection from infection and viral replication in an NHP model of SARS-CoV-2 infection

**Fig. 5:**
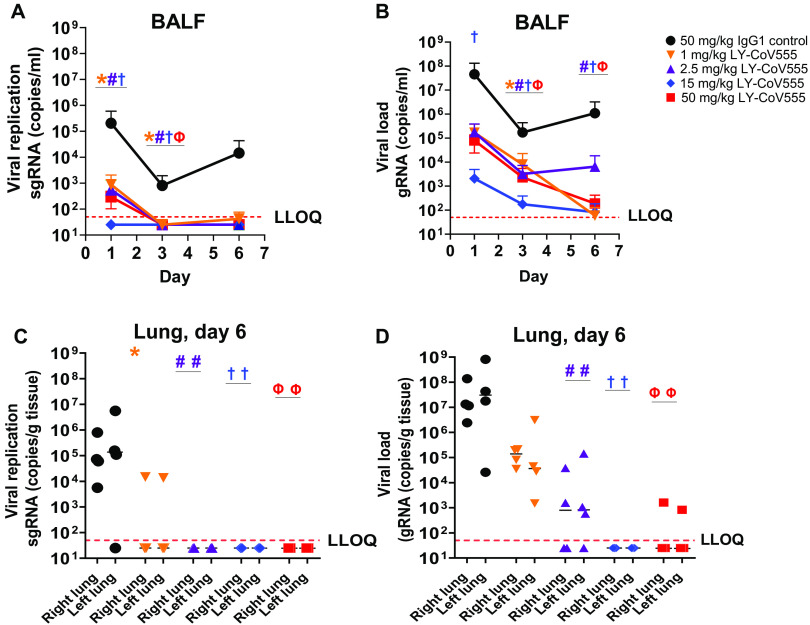
LY-CoV555 pretreatment reduces viral replication and load in the lower respiratory tract of rhesus macaques challenged with SARS-CoV-2. Rhesus macaques (*n* = 3 or 4 per group) received LY-CoV555 (1, 2.5, 15, or 50 mg/kg) as a single intravenous dose 24 hours before SARS-CoV-2 inoculation. (**A**) sgRNA (viral replication) and (**B**) gRNA (viral load) were assessed by qRT-PCR in BALF over the course of 6 days after inoculation. (**C**) sgRNA (viral replication) and (**D**) gRNA (viral load) were assessed by qRT-PCR in lung tissue on day 6. Values represent the mean and SEM for three or four animals (A to C) or the mean of three or four animals (D). Samples below the lower limit of quantification (LLOQ) were designated a value of ½ LLOQ for plotting. LLOQ = 50 copies for genomic or subgenomic mRNA. Statistical testing results comparing treatment to the corresponding IgG1 control are provided in table S6. * denotes *q* value < 0.05, 1 mg/kg; # denotes *q* value < 0.05, 2.5 mg/kg; † denotes *q* value < 0.05, 15 mg/kg; and Ф denotes *q* value < 0.05, 50 mg/kg. BALF, bronchoalveolar lavage; gRNA, genomic RNA; qRT-PCR, quantitative real-time polymerase chain reaction; sgRNA, subgenomic RNA.

**Fig. 6:**
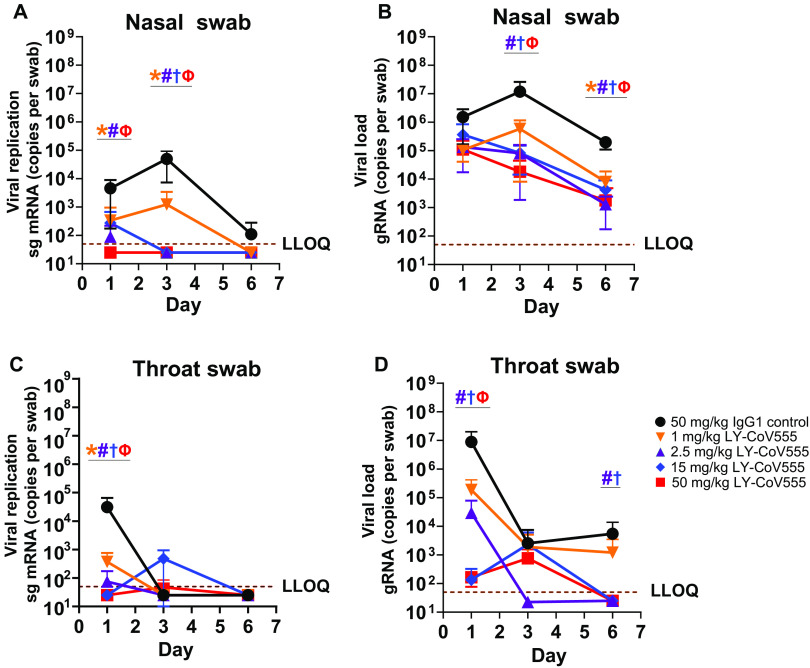
LY-CoV555 pretreatment reduces viral replication and load in the upper respiratory tract of rhesus macaques challenged with SARS-CoV-2. Rhesus macaques (*n* = 3 or 4 per group) received LY-CoV555 (1, 2.5, 15, or 50 mg/kg) as a single intravenous dose 24 hours before viral challenge. (**A**) sgRNA (viral replication) and (**B**) gRNA (viral load) were assessed by qRT-PCR in nasal swabs over 6 days after SARS-CoV-2 inoculation. (**C**) sgRNA (viral replication) and (**D**) gRNA (viral load) were assessed by qRT-PCR in throat swabs over 6 days after SARS-CoV-2 inoculation. Values represent the mean ± SEM for three or four animals at indicated time points. Samples below the lower limit of quantification (LLOQ) were designated a value of ½ LLOQ for plotting. LLOQ = 50 copies for gRNA or sgRNA. Statistical testing results comparing treatment to the corresponding IgG1 control are provided in table S6. * denotes *q* value < 0.05, 1 mg/kg; # denotes *q* value < 0.05, 2.5 mg/kg; † denotes *q* value < 0.05, 15 mg/kg; and Ф denotes *q* value < 0.05, 50 mg/kg. gRNA, genomic RNA; qRT-PCR, quantitative real-time polymerase chain reaction; sg mRNA, subgenomic RNA.

To assess the ability of LY-CoV555 to protect from viral challenge, we used a rhesus macaque model of SARS-CoV-2 infection (*15*). LY-CoV555 antibody was administered intravenously (IV) to rhesus macaques at a dose of 1, 2.5, 15, or 50 mg/kg 24 hours before virus challenge. Control animals received a control IgG1 antibody (50 mg/kg) IV. The LY-CoV555 doses were chosen to provide a range of serum antibody concentrations and inform subsequent clinical dosing. After inoculation, respiratory and clinical signs of disease in the macaques were limited. Mild lobar congestion and hyperemia were observed macroscopically across control and treated groups, suggestive of either interstitial or bronchopneumonia (table S5). Subgenomic RNA (sgRNA) and viral genomes (gRNA), indicative of active viral replication (*15*), were detectable in bronchoalveolar lavage fluid (BALF), throat swabs, and nasal swabs for all control animals after intranasal and intratracheal inoculation with SARS-CoV-2 (Figs. 5 and 6).

Prophylactic administration of LY-CoV555 resulted in decreases in viral replication and viral load as evaluated by sgRNA and gRNA, respectively, in the BALF and lung tissue from the lower respiratory tract after SARS-CoV-2 inoculation (Fig. 5 and table S6). In the BALF, reductions of 10^2^ to 10^5^ copies per milliliter in viral replication and load were observed compared to controls across days 1, 3, and 6, with significant reductions in viral replication (1, 2.5, and 15 mg/kg doses; Fig. 5A) and load (15 mg/kg dose; Fig. 5B) on day 1 and at all doses on day 3 relative to control IgG1-treated animals (*q* < 0.05). In LY-CoV555–treated animals, viral replication in BALF was undetectable by day 3 at all doses (Fig. 5A). Consistent with BALF on day 6, no viral replication was observed in lung tissue harvested at necropsy in the 2.5, 15, and 50 mg/kg dose groups, demonstrating a significant reduction (*q* value < 0.05) compared to control animals (Fig. 5C and table S6). Viral loads in the BALF and lung on day 6 were significantly reduced (*q* value < 0.05) at the 2.5, 15, and 50 mg/kg doses (Fig. 5, B and D, and table S6).

LY-CoV555 also provided protection in the upper respiratory tract, whereby viral replication was significantly reduced in the nose (1, 2.5, and 50 mg/kg doses; Fig. 6A and table S6) as compared to IgG1 control animals on day 1 (*q* value < 0.05). By day 3, virus replication was undetectable in the nose (<50 copies per swab) at doses of 2.5, 15, and 50 mg/kg (Fig. 6A and table S6). There was also significant reduction in gRNA (*q* value < 0.05) at the 2.5, 15, and 50 mg/kg doses on days 3 and 6 as compared to control animals (Fig. 6B and table S6). On day 1, viral replication was also significantly reduced in the throat at doses of 1, 2.5, 15, and 50 mg/kg (Fig. 6C and table s6) and in gRNA (*q* value < 0.05) at doses of 2.5, 15, and 50 mg/kg (Fig. 6D and table S6).

### Serum concentrations of LY-CoV555 required for protection from SARS-CoV-2 infection

**Fig. 7:**
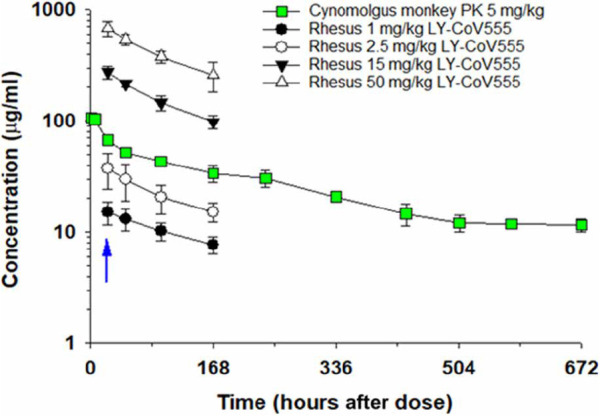
Determination of serum concentrations of LY-CoV555 in a cynomolgus monkey PK study and in rhesus macaques during SARS-CoV-2 infection. In the PK study, female cynomolgus monkeys received LY-CoV555 (5 mg/kg) as a single intravenous dose, and serum samples were collected through 672 hours after administration. Samples were analyzed using a human IgG ELISA or a ligand-capture LC-MS assay, which provided comparable results. Data points represent mean ± SD of determination from three animals. Cynomolgus monkey PK data are represented by green squares. In the rhesus macaque challenge experiments, animals (*n* = 3 or 4 per group) were administered LY-CoV555 (1, 2.5, 15, or 50 mg/kg) as a single intravenous dose, and serum samples were collected on study day −1 (predose) and days 0, 1, 3, and 6 (24, 48, 96, and 168 hours after intravenous dosing). Data points represent the mean ± SD for three or four animals. The blue arrow refers to viral challenge in rhesus macaque study on study day 0 (24 hours after intravenous administration of LY-CoV555).

Before initiating the rhesus macaque challenge model, a pharmacokinetic (PK) study was conducted in cynomolgus monkeys to confirm the anticipated characteristics of LY-CoV555 dosed via the intravenous route. LY-CoV555 administration resulted in sustained serum concentrations after intravenous dosing, with a half-life of elimination of 13 days, and a clearance of 0.22 ml hour^−1^ kg^−1^, consistent with expected PK for human IgG1 in an NHP model (Fig. 7) (*28*, *29*). Serum concentrations of LY-CoV555 were evaluated during rhesus macaque prophylactic SARS-CoV-2 challenge experiments. Serum LY-CoV555 in the rhesus macaques was dose proportional with the cynomolgus monkey PK (Fig. 7). Mean serum concentrations of LY-CoV555 on the day of viral challenge were 15 ± 3, 38 ± 14, 276 ± 37, and 679 ± 101 mg/ml at doses of 1, 2.5, 15, and 50 mg/kg, respectively (table S7). On the basis of the maximal infection protection provided at doses of 2.5 mg/ml and above, the 38 mg/ml serum concentration at the time of viral challenge provides a target for protective drug concentrations in this model. Given the substantial viral inoculum, this value may overestimate serum concentrations needed to provide protection in community-acquired infections.

In addition, BALF concentrations of LY-CoV555 were determined in rhesus macaque prophylactic SARS-CoV-2 challenge experiments (table S8). BALF concentrations of LY-CoV555, along with BALF urea concentrations, were used to estimate lung epithelial lining fluid (ELF) concentrations of LY-CoV555 using a previously described method (*30*). Median BALF concentrations and estimated ELF concentrations generally increased with increasing dose when comparing the 1, 2.5, and 15 mg/kg doses of LY-CoV555. However, BALF and ELF concentrations in the 50 mg/kg groups (treatment and controls) did not show dose-related increases compared to the 15 mg/kg dose. Median estimated ELF concentrations as a percentage of serum concentration ranged from 2 to 24% (table S8). These values are in the range of nasal ELF concentrations previously reported for another therapeutic antibody (*31*).

## DISCUSSION

This study describes the rapid identification and characterization of a potent anti-spike neutralizing antibody, LY-CoV555, derived from PBMCs isolated from a patient after recovery from COVID-19. After antibody screening, LY-CoV555 demonstrated greater neutralization potency of SARS-CoV-2 compared to the other antibodies found from this patient (*32*). LY-CoV555 was found to have high-affinity RBD-binding and ACE2-blocking properties, which translated to high neutralization potency owing to its SARS-CoV-2 spike protein–binding properties. In both in vitro assays with full virus and an NHP model of SARS-CoV-2 infection, LY-CoV555 displayed high protection potency, supporting its clinical development and testing as a therapeutic for the treatment and prevention of COVID-19.

Our NHP challenge study also provides evidence that neutralizing antibodies have potential as an important countermeasure for preventing COVID-19 disease. Antibody treatment may reduce virus replication in the upper airway, thus decreasing viral shedding and transmission after treatment. Overall, we show dose-related reductions in gRNA and sgRNA in the upper and lower respiratory tracts with maximal protection observed at doses of 2.5 mg/kg and above. Given the robust nature and route of administration of the viral inoculum in this model, we hypothesized that modest doses of LY-CoV555 could provide substantial clinical efficacy.

Serum LY-CoV555 concentrations in the rhesus macaque model were dose responsive and demonstrated sustained exposure as expected for a human IgG1 antibody in an NHP model. Maximal inhibition of viral replication across the upper and lower respiratory tract was observed at doses of 2.5 mg/kg and above, associated with a mean serum concentration of 37.5 mg/ml at the time of infection. Median LY-CoV555 concentrations estimated in ELF fluid were 2 to 24% of serum concentrations, which was in general agreement with literature reports of antibody distribution to ELF fluid (*31*, *33*). These data were also in the range of a literature-based physiologically based PK model–derived value of 6.5% used in clinical modeling and simulation to support study design. At all of the LY-CoV555 doses tested in this study, median lung ELF concentrations exceeded the effective concentration for 90% inhibition (EC_90_) for SARS-CoV-2 virus neutralization, which is consistent with the observed reductions in viral replication in the BALF and lung tissue across the dose range tested, even at substantially higher (10-fold) viral challenge doses relative to vaccine studies in this rhesus macaque model (*18*). ELF fluid concentrations were not evaluated in the nose and throat. As compared to the lung and throat, the delayed impact on viral loads in nasal swabs could reflect differential distribution of antibody into the nasal ELF.

The PBMC sample, from which LY-CoV555 was derived, was collected about 20 days after symptom onset. This is an early time point in the disease course and in the immune response to viral infection. Despite the lack of substantial somatic mutation of antibodies, as evidenced by the high sequence similarity to germline, we were able to identify several antibodies to the spike protein capable of neutralizing viral infection in ACE2-bearing cells, including some that did not directly block ACE2 engagement. Comparison of similar discovery approaches using samples from convalescent patients suggests that the collection of antibodies derived from this patient may have had relatively few mAbs with potent neutralizing activity (*7*, *12*). Several factors might be responsible for these differences, including the patient’s immune status and disease severity, the relatively early collection of the sample used for antibody discovery, the depth of screening and robust assays afforded by our microfluidic platform, the availability of structurally defined protein probes, or the very broad approach taken with respect to antigenic diversity. Nonetheless, our approach identified a highly potent neutralizing antibody from a patient sample that had been characterized as having limited antibody response and neutralizing capacity (*32*).

The importance of prototype pathogen preparedness was demonstrated by the ability to rapidly design and produce protein for B cell probes based on prior work defining the structure and stabilization strategy for the betacoronavirus spike protein (*34*). The resulting speed at which this drug discovery and development effort proceeded (fig. S7), with progression to human treatment only 90 days after the initiation of antibody screening, was due to advanced discovery and characterization platforms and pre-established public-private partnership.

Both monotherapy and antibody combinations are being explored clinically (*14*). Monotherapy with a single potent antibody represents a pragmatic option to combat an ongoing pandemic with a virus that causes an acute, self-limited infection. For both respiratory syncytial virus (RSV) and Ebola virus, there are clinical precedents for monotherapy prophylaxis or treatment, respectively, with potent neutralizing mAbs (*35*). Specifically, in the case of RSV, infants have been effectively treated with palivizumab since its introduction in 1996 (*36*). As therapy, neutralizing mAbs, such as LY-CoV555, could supplement an ongoing endogenous adaptive immune response to the virus, with its own diverse polyclonal antibodies in addition to other responses such as expansion of specific CD4^+^ and CD8^+^ T cell populations (*37*). This endogenous polyclonal antibody repertoire, which will have neutralizing activity against diverse epitopes, supplemented with virus-specific T cell responses, should minimize the likelihood that escape mutants will arise during acute infection.

As expected, the spike protein, and the RBD in particular, has been susceptible to mutations due to its pivotal role in the infection process. There have been a number of variants emerging recently that contain an N501Y mutation, which is associated with increased transmissibility. This mutation is found in the lineages B.1.1.7 and B.1.351, which were found in the United Kingdom and South Africa, respectively (*38*, *39*). On the basis of the structure of the LY-CoV555:RBD complex, N501Y does not reside within the epitope for this antibody. However, B.1.351 includes two other mutations at important residues in the RBD, K417N and E484K, of which only E484 falls within the epitope of LY-CoV555 (*39*). As would be expected, mutations at residues within the epitope of LY-CoV555 have the potential to affect the binding and function, whereas residues outside the epitope do not. For example, low-frequency mutations that have been observed in GISAID (global initiative on sharing all influenza data) at positions V483, E484, F490, and S494 either decrease or eliminate binding and function, whereas mutations at V367, K417, S477, and N501 have no effect (*40*). Although this paper focuses on the rapid identification and preclinical characterization of one mAb, human studies are evaluating both single and mAb combinations using LY-CoV555 and led to the Emergency Use Authorizations of both bamlanivimab alone and together with etesevimab (*41*, *42*).

This study focuses on the identification and characterization of a single mAb that binds to the RBD of the SARS-CoV-2 spike protein. This was a consequence, in part, of the very limited number of the discovered antibodies exhibiting potent neutralization. These results indicate that one limitation to the approach taken was due to the timing of the patient sample relative to infection; at this early point in the evolution of the patient’s immune response, very deep screening was required to identify potent neutralizing antibodies. Future pandemic response efforts might take into consideration this aspect and use an approach that balances timing with the ability to identify greater numbers of highly potent antibodies to enable a rapid discovery of multiple antibodies for use in cocktails.

A limitation of the animal model studies is the focus on testing LY-CoV555 in a prophylactic setting in an NHP model that does not recapitulate the full disease physiology of COVID-19 in humans. In addition, we did not study the therapeutic effect of LY-CoV555 in this animal or other animal models. We focused on prophylaxis for two primary reasons: first, the NHP model is better suited to test prevention of disease; and second, because of the rapid speed of development of LY-CoV555, efficacy in the therapeutic setting was already being explored clinically at the time of these experiments. Subsequent clinical trial results with LY-CoV555 administered as a treatment to patients infected with SARS-CoV-2-and with mild to moderate disease demonstrated reduction in viral load, reduction in COVID-19 symptoms, and an approximate threefold decreased rates of hospitalization in the 700-, 2800-, and 7000-mg dose groups relative to placebo, indicating activity as a treatment and the potential for efficacy at lower doses (*22*). In addition, we felt that it was important to understand the efficacy dose response, especially with respect to blood and BALF concentrations of LY-CoV555, as they relate to preventive efficacy. This could be studied in this animal model. This study also informs subsequent use in a postexposure prophylactic setting as is being explored clinically (NCT04497987). Last, other efforts had already demonstrated that antibodies effective in prophylaxis were also effective in treatment in multiple animal models, albeit with different potency (*13*, *43*).

LY-CoV555 was developed as a therapeutic antibody specifically to treat COVID-19. The treatment quickly entered clinical testing (*44*, *45*) and demonstrated clinical efficacy (*22*), and gain Emergency Use Authorization (*42*). LY-CoV555 is presently under clinical evaluation for the treatment and prevention of COVID-19 (NCT04411628, NCT04427501, NCT04497987, NCT04501978, NCT04518410, and NCT04634409) in various clinical settings. Overall, the identification and characterization of LY-CoV555 points to the feasibility of strategies to rapidly identify neutralizing human mAbs as part of an initial response to an evolving pandemic that can complement population-scale vaccination, provide immediate passive immunity, and provide protection for vulnerable populations.

## MATERIALS AND METHODS

### Study design

This study was designed to identify SARS-CoV-2 neutralizing antibodies from a convalescent patient with COVID-19. This objective was addressed by first conducting a detailed screening of antibodies produced from patient-derived PBMCs to identify high-affinity SARS-CoV-2 spike protein binding and was followed by a variety of high-throughput binding characterization experiments to identify SARS-CoV-2 neutralizing antibodies. All in vitro characterization of binding properties and viral infection neutralization were carried out in a screening fashion, with *n* = 1, and the number of technical replicates as described in the associated figure legends. For in vivo characterization of the ability of LY-CoV555 to provide protection from SARS-CoV-2 infection, animals were randomized to dose groups to achieve a similar average age for each group. The number of animals in each dose group, timing of drug administration, and virus inoculation were informed by available data regarding the rhesus macaque model of SARS-CoV-2 infection (*15*). In the cynomolgus monkey PK study, naïve monkeys were selected from the PK colony to minimize the potential impact of antihuman antibodies on the PK profile. With respect to the number of animals per group and the duration of sample collection, the PK study leveraged a standard study design. Researchers were blinded to the identity of antibodies where possible. All data points were included in the analyses, and no outliers were excluded.

### Single-cell screening and recovery

A blood sample from a 35-year-old individual hospitalized with severe COVID-19 disease was obtained mid-February 2020, about 20 days after the onset of symptoms. PBMC samples were collected under institutional review board–approved protocols as part of the Hospitalized and Ambulatory Adults with Respiratory Viral Infections study at the University of Washington (protocol no. STUDY00000959) and Vaccine Research Center (VRC), National Institute of Allergy and Infectious Diseases (NIAID), and National Institutes of Health (NIH) (protocol-VRC400, NIH-07IN194). Cells were thawed, activated in culture to generate memory B cells, and enriched for antibody-secreting B cells before injection into AbCellera’s microfluidic screening devices with either 91,000 or 153,000 individual nanoliter-volume reaction chambers (*46*–*54*). Single cells secreting target-specific antibodies were identified and isolated using two assay types (*55*): a multiplexed bead assay using multiple optically encoded beads, each conjugated to the soluble prefusion-stabilized spike of either SARS-CoV-1 or SARS-CoV-2 spike with T4-foldon domain, 3C protease cleavage site, 6× His-tags, and twin-strep tags (*34*) or negative controls [bovine serum albumin (BSA) His-tag and T4 FoldOn trimerization domain], and a live-cell assay using passively dyed suspension-adapted Chinese hamster ovary (CHO) cells transiently transfected to surface-express full-length SARS-CoV-2 spike protein (GenBank ID MN908947.3) with a green fluorescent protein (GFP) reporter and nontransfected cells as a negative control. Beads or cells were flowed onto microfluidic screening devices and incubated with single antibody-secreting cells, and mAb binding to cognate antigens was detected via a fluorescently labeled antihuman IgG secondary antibody. Positive hits were identified using machine vision and recovered using automated robotics-based protocols.

### Single-cell sequencing, bioinformatic analysis, and cloning

Single-cell polymerase chain reaction (PCR) and NGS (MiSeq, Illumina) were performed using automated workstations (Bravo, Agilent) and custom molecular biology protocols for the recovery of paired heavy- and light-chain sequences. Sequencing data were analyzed using a custom bioinformatics pipeline to yield paired heavy- and light-chain sequences for each recovered antibody-secreting cell (*56*). Each sequence was annotated with the closest germline [V(D)J] genes, degree of somatic hypermutation, and potential sequence liabilities. Antibodies were considered members of the same clonal family if they shared the same inferred heavy and light V and J genes and had the same CDR3 length. The variable [V(D)J] region of each antibody chain was PCR-amplified and inserted into expression plasmids using a custom, automated high-throughput cloning pipeline. Plasmids were verified by Sanger sequencing to confirm the original sequence previously identified by NGS. Antibodies were recombinantly produced by transient transfection in either human embryonic kidney 293 or CHO cells as described in Supplementary Materials and Methods.

### Binding validation and analysis

Recombinant antibodies were confirmed to bind screening targets using two assay types via high-throughput flow cytometry. In a multiplexed bead–based assay, optically encoded beads were conjugated to one of the following unique antigens: spike proteins of SARS-CoV-2, MERS-CoV, SARS-CoV-1, human coronavirus (HKU1-CoV), bat SARS-like WIV1 coronavirus, or the S1 subunit of SARS-CoV-2 spike protein. Purified antibodies were incubated with target-conjugated and negative control BSA His-tag and T4 FoldOn–conjugated beads at either 50, 10, or 2 nM antibody concentration for 30 min at room temperature (RT). In a live cell–based assay, full-length spike protein sequences of either the wild type or mutants V367F, V483A, and D614G of SARS-CoV-2 with GFP inserts were transiently transfected into CHO cells (MaxCyte STX Scalable Transfection System). Full-length native conformation spike protein expression was confirmed via GFP detection, flow cytometry–detected binding to S1 and S2 subunit–specific benchmark antibodies, and Western blot. Purified antibodies were incubated with target-expressing cells and nontransfected control cells at 50, 10, or 2 nM antibody concentration for 30 min at 4°C. Beads or cells were washed, and binding was detected using a fluorescently labeled antihuman IgG secondary antibody. Fluorescence was measured using high-throughput plate-based flow cytometry. Benchmark antibodies previously identified from SARS-CoV-1 convalescent patient samples and cross-reactive to SARS-CoV-2 spike protein were used as positive controls; human IgG isotype and an irrelevant antibody specific to HIV, VRC01, were used as negative controls. Median fluorescence intensity of each antibody was normalized over the median fluorescence intensity of the human isotype, with signals greater than 5-fold over isotype control (and less than 2.5-fold binding to negative controls) considered as specific binding.

### SPR binding experiments

All high-throughput SPR binding, epitope binning, and ACE2 competition experiments were performed on a Carterra LSA instrument equipped with an HC-30M chip type (Carterra-bio) using a 384-ligand array format. For all experiments, antibodies were coupled to the HC-30M chip: The chip surface was first activated by flowing a freshly prepared 1:1:1 activation mix of 100 mM MES (pH 5.5), 100 mM sulfo–*N*-hydroxysuccinimide, and 400 mM 1-ethyl-3-(3-dimethylaminopropyl)carbodiimide for 7 min, and antibodies diluted to either 10 or 1 μg/ml in 10 mM NaOAc (pH 4.25) buffer + 0.01% Tween were injected and printed simultaneously onto the chip surface for 10 min by direct coupling. The chip surface was quenched by flowing 1 M EtOHamine for 7 min, followed by two wash steps of 15 s each in 25 mM MES (pH 5.5) buffer. Relevant benchmarks and negative control antibodies (HIV VRC01, mouse FoldOn 8203-C1, and rabbit His-tag PA1-983) were also printed on the chip surface.

For binding kinetics and affinity measurements, a threefold dilution series of the antigen of interest, starting at 300 nM in HEPES-buffered saline containing 0.05% Tween 20 and 3 mM EDTA (HBSTE) + 0.1% BSA running buffer, was sequentially injected onto the chip surface. For each concentration, the antigen was injected for 5 min (association phase), followed by running buffer injection for 15 min (dissociation phase). Two regeneration cycles of 15 s were performed between each dilution series by injecting Pierce IgG elution buffer (Thermo Fisher Scientific) + 1 M NaCl on the chip surface. The data were analyzed using the Carterra Kinetics analysis software using a 1:1 Langmuir binding model to determine apparent association (*k*_a_) and dissociation (*k*_d_) kinetic rate constants and binding affinity constants (*K*_d_).

For epitope binning experiments, antibodies coupled to the chip surface were exposed to various antibody:antigen complexes. Samples were prepared by mixing each antibody in 10- to 20-fold molar excess with antigen (1:1 freshly prepared mix of 400 nM antibody and 40 nM antigen, both diluted in 1× HBSTE + 0.1% BSA running buffer). Each antigen-antibody premix was injected sequentially over the chip surface for 4 min (association phase to ligand printed onto chip previously), followed by a running buffer injection for 2 min (dissociation phase). Two regeneration cycles of 15 s were performed between each premix sample by injecting 10 mM glycine (pH 2.0) onto the chip surface. An antigen-only injection (20 nM concentration in running buffer) was performed every 8 cycles. The data were analyzed using the Carterra Epitope analysis software (version 1.2.0.1960) for heatmap and competition network generation. Analyte binding signals were normalized to the antigen-only binding signal, such that the antigen-only signal average is equivalent to one relative unit (RU). A threshold window ranging from 0.9 to 1.1 RU was used to classify analytes into three categories: blockers (binding signal under the lower limit threshold), sandwichers (binding signal over the higher limit threshold), and ambiguous (binding signal between limit thresholds). Antibodies with low coupling to the chip, poor regeneration, or absence of self-blocking were excluded from the binning analysis. Like-behaved antibodies were automatically clustered to form a heatmap and competition plot.

For ACE2 competition experiments, antibodies coupled to the chip were exposed to spike protein:ACE2 complex; 20 nM SARS-CoV-2 spike protein was premixed with 200 nM His-tagged ACE2 (ACE2-His) diluted in HBSEP+ with 0.5 M NaCl, 1% BSA, 1× dextran, and heparin (2 mg/ml), and incubated for about 12 hours. The complex of spike protein/ACE2-His was then tested for binding to immobilized antibodies on the prepared HC30M chip, with association for 5 min and dissociation for 1 min. Regeneration was performed in 20 mM glycine (pH 2.0) with 1 M NaCl for 30 s twice.

### Negative-stain electron microscopy

SARS-CoV-2 spike ectodomain was diluted to 0.04 mg/ml in 2 mM tris (pH 8.0), 200 mM NaCl, and 0.02% NaN_3_ (dilution buffer) in the presence of 10-fold excess Fab and incubated on ice for 10 s. CF400-Cu grids (Electron Microscopy Sciences) were plasma-cleaned for 30 s in a Solarus 950 plasma cleaner (Gatan) with a 4:1 ratio of O_2_/H_2_. A volume of 4.8 μl of the protein sample was applied to the grid and allowed to incubate for 30 s. The grid was then washed twice with dilution buffer before staining with methylamine tungstate (NANO-W, Nanoprobes). Grids were imaged using a FEI Talos TEM (Thermo Fisher Scientific) and a Ceta 16M detector. Micrographs were collected manually using TIA v4.14 software at a magnification of ×92,000, corresponding to a pixel size of 1.63 Å/pixel. Contrast transfer function (CTF) estimation and particle picking were performed in cisTEM. A 2D classification was performed in either cisTEM (*57*) or cryoSPARC v2.15.10 (*58*), and antibody initio reconstruction and refinement of 3D maps were performed in cryoSPARC.

### Cryo–electron microscopy

A purified, prefusion-stabilized SARS-CoV-2 spike variant, HexaPro (*59*), at 0.2 mg/ml was complexed with 1.3-fold molar excess of LY-CoV555 Fab in 2 mM tris (pH 8), 200 mM NaCl, and 0.02% NaN_3_ for 5 min on ice. Three microliters of protein complex was deposited on an UltrAuFoil 1.2/1.3 grid (Electron Microscopy Sciences), which had been plasma-cleaned for 2 min using a Gatan Solarus 950 with a 4:1 O_2_:H_2_ ratio. The grid was then plunge-frozen in liquid ethane using a Vitrobot Mark IV (Thermo Fisher Scientific) set to 100% humidity and 22°C, with a blot time of 5 s and a blot force of −4. Data were collected on a Titan Krios operating at 300 kV and equipped with a K3 detector using a magnification of 22,500×, resulting in a pixel size of 1.045 Å. A total of 30 frames were collected for each micrograph, with defocus values ranging from −0.8 to −2.8 μm, a total exposure time of 4.5 s, and a total electron dose of ~32.7 e^−^/Å^2^. A full description of the data collection parameters can be found in table S9 and fig. S6. Motion correction, CTF estimation, and particle picking were performed in Warp (*60*). Particles were subsequently transferred to cryoSPARC v2.15.10 (*58*) for 2D classification and 3D reconstruction. The refined map was then subjected to local *B*-factor sharpening using LocalDeBlur (*61*). Model building and refinement were subsequently performed using Coot, Phenix, and ISOLDE (*62*–*64*).

### Protein crystallography

For protein crystallography, an isolated RBD (using residues 329 to 527) was fused to a 6× His-tag at the C terminus, expressed in CHO cells, enzymatically deglycosylated using endoglycosidase-H (Endo-Hf, New England Biolabs), and purified by cation exchange chromatography. The Fab portions of selected antibodies, containing mutations in the constant region known to encourage crystallization (*65*), were expressed in CHO cells and purified. The Fab-RBD complexes were prepared by mixing the components with a 20% excess of the RBD and then the complex was purified from the excess RBD by size-exclusion chromatography. Fab-RBD complexes (about 12 mg/ml) were crystallized by vapor diffusion sitting drops. Crystals of complexes formed within 1 to 2 days and were harvested on the third day. Crystals were flash-frozen in liquid nitrogen after 1-min incubation in cryoprotectant solution containing 25% glycerol in mother liquor: LY-CoV555 Fab-RBD complex was crystallized using 100 mM sodium acetate (pH 4.6) and 20% PEG 10K; the 481CK Fab-RBD complex was crystallized using 100 mM tri-sodium citrate (pH 5.8), 14% PEG 4K, and 10% 2-propanol; and the 488 CK Fab-RBD complex was crystallized using 100 mM HEPES (pH 7.7), 8% PEG 3350, and 200 mM l-proline.

Diffraction data were collected at Lilly Research Laboratories Collaborative Access Team and beamline at Sector 31 of the Advanced Photon Source at Argonne National Laboratory. Crystals stored in liquid nitrogen were mounted on a goniometer equipped with an Oxford Cryosystems cryostream maintained at a temperature of 100 K. The wavelength used was 0.9793 Å, collecting 900 diffraction images at a 0.2° oscillation angle and 0.12-s exposure time on a Pilatus3 S 6M detector at a distance of 392 mm.

The diffraction data were indexed and integrated using autoPROC (*66*)/XDS (*67*) and merged and scaled in AIMLESS (*68*) from the CCP4 suite (*69*). Nonisomorphous data readily yielded initial structures by molecular replacement using for the Fab portion crystal structures from the proprietary Eli Lilly structure database and for the SARS-CoV-2 spike RBD from the public domain structure with the access code 6yla (*70*). The initial structure coordinates for each dataset were further refined using Refmac5 (CCP4), applying isotropic temperature factors. Model building was performed with Coot (CCP4) and final structure validation was performed with MolProbity (*71*) and CCP4 validation tools. Table S10 presents the crystallographic data statistics. Protein coordinates and structure factors have been deposited with the Protein Data Bank under the access codes 7KMG, 7KMH, and 7KMI for Ab169 (LY-CoV555), Ab133, and Ab128, respectively.

### Pseudotyped neutralization assay for mAb screen

SARS-CoV-2 spike pseudotyped lentiviruses that harbor a luciferase reporter gene were produced, and neutralization assay was performed as described previously (*72*, *73*). Pseudovirus was produced by cotransfection of 293T cells with plasmids encoding the lentiviral packaging and luciferase reporter, a human transmembrane protease serine 2 (TMPRSS2), and SARS-CoV-2 S (Wuhan-1, GenBank no. MN908947.3) genes. Forty-eight hours after transfection, supernatants were harvested, filtered, and frozen. For initial screening neutralization assay, four dilutions of mAbs (10, 1, 0.1, and 0.01 μg/ml) were mixed with titrated pseudovirus, incubated for 45 min at 37°C, and added to preseeded ACE2-transfected 293T cells (either transiently or stably transfected) in 96-well white/black Isoplates (PerkinElmer). After 2 hours of incubation, wells were replenished with 150 μl of fresh medium. Cells were lysed 72 hours later and luciferase activity (relative light unit) was measured. Percent neutralization was calculated relative to pseudovirus-only wells.

### Neutralization activity of antibodies against authentic SARS-CoV-2

Authentic SARS-CoV-2 neutralization activity of the discovered antibodies was measured by detecting the neutralization of infectious virus in cultured Vero E6 cells [African green monkey kidney; American Type Culture Collection (ATCC), #CRL-1586]. These cells are known to be highly susceptible to infection by SARS-CoV-2. Cells were maintained according to standard ATCC protocols. Briefly, Vero E6 cells were grown in minimal essential medium (MEM) supplemented with 10% heat-inactivated fetal bovine serum (FBS), 2 mM l-glutamine, and 1% of MEM nonessential amino acid (NEAA) solution (Thermo Fisher Scientific, #MT25025CI). Cell cultures were grown in 75- or 150-cm^2^ flasks at 37°C with 5% CO_2_ and passaged two to three times per week using trypsin-EDTA. Cell cultures used for virus testing were prepared as subconfluent monolayers. All incubations containing cells were performed at 37°C with 5% CO_2_.

### Production of virus inocula

Immunofluorescent and plaque reduction assays were conducted using virus produced by infecting cultured Vero E6 cells with the SARS-CoV-2 clinical isolate USA/WA-1/2020 [Biodefense and Emerging Infections Research Resources Repository (BEI) resources number NR52281] or the INMI-1 isolate (European Virus Archive–Global, ref. no. 008V-03893) and incubating at 37°C until cytopathology is evident (typically 48 to 72 hours). Expansion was limited to one to two passages in cell culture to retain integrity of the original viral sequence. The virus stock was quantified by standard plaque assay, and aliquots were stored at −80°C. A freshly thawed aliquot was used for each neutralization experiment.

### Virus neutralization detected by immunofluorescence

Virus infectivity assays were conducted in 96-well tissue culture plates. Vero E6 cells were seeded at a density of 8 × 10^4^ cells/cm^2^ and incubated overnight to a confluency of about 95%. Serial dilutions of antibodies or positive control polyclonal serum from a convalescent patient with SARS-CoV-2 were prepared in DMEM (Dulbecco’s modified essential medium; Gibco, #11965-092) supplemented with 1% NEAA and 10 mM HEPES. Virus stock [prepared for a final concentration of 18 to 20 TCID_50_ (median tissue culture infectious dose) per well] was added to each dilution of antibody and incubated for 1 hour. Virus with no antibody and no-virus wells served as controls. Incubated samples were inoculated onto Vero E6 cells at a final volume of 100 μl, and plates were incubated for 24 hours. To detect virus replication, the inoculum was removed, and monolayers were fixed in 10% formalin solution (4% active formaldehyde) for 1 hour at RT. Background staining was quenched by adding 50 mM NH_4_Cl to cells and rocking for 10 min at RT, followed by washing. Cells were permeabilized with 0.1% Triton-X 100 (by rocking at RT for 10 min), washed three times with Dulbecco’s phosphate-buffered saline (DPBS), and nonspecific antibody binding was blocked with 1% BSA. Mouse anti–SARS-CoV-2 nucleoprotein antibody (1 C7C7, a gift from T. Moran, Icahn School of Medicine at Mount Sinai), diluted at 1:1000 in DPBS with 1% BSA, was added to each well and incubated overnight at 4°C. After washing, cells were stained with goat anti-mouse Alexa Fluor plus 647 antibody (Thermo Fisher Scientific, #A32728; green dye) and 4′,6-diamidino-2-phenylindole (dihydrochloride; Thermo Fisher Scientific, #62247; blue dye) by incubating for 1 hour at 37°C. Images were collected using a CellInsight CX7 with the 4× objective covering the entire well. The percentage of infected cells per well relative to the uninfected and no-antibody controls was analyzed using the instrument’s “Target Activation” analysis protocol.

### Virus neutralization detected by luciferase reporter

Luciferase assays were performed using a molecular complementary DNA clone of a SARS-CoV-2 isolate (USA/WA-1/2020) in which a nonessential gene (ORF7) was replaced by the NanoLuc luciferase reporter gene (Promega), as previously described for SARS-CoV-1 and MERS-CoV (*74*). Virus infectivity assays were conducted in 96-well tissue culture plates. Vero E6 cells were seeded at a density of 2 × 10^4^ cells per well in DMEM supplemented with 10% FBS (DMEM/FBS) and incubated for 15 to 24 hours. The next day, serial dilutions of antibodies or human IgG1 isotype control were prepared in DMEM/FBS. The SARS-CoV-2–NanoLuc inoculum was diluted in DMEM/FBS, mixed with an equal volume of diluted antibody to produce a final virus titer of 140 plaque-forming units (PFU) per well, and incubated for 1 hour. After removing the culture medium from the plated Vero E6 cells, the virus-antibody solution was inoculated onto duplicate wells and incubated for 48 hours. Following standard protocols as recommended by the vendor, Nano-Glo reagent (Promega, #N1110) was added and luciferase activity was quantified on a SpectraMax plate reader (Molecular Devices).

### Virus neutralization detected by plaque reduction

Plaque reduction assays were performed in six-well plates. Vero E6 cells were seeded at a concentration of about 10^6^ cells per well and grown overnight to reach 95% confluency. The next day, serial threefold dilutions of antibody were prepared in Eagle’s MEM, mixed with about 100 PFU of SARS-CoV-2, and incubated for 1 to 2 hours. The antibody/virus mixtures were inoculated directly onto the cells and allowed to adsorb for 1 hour, with rocking at 15-min intervals. An overlay medium composed of 1.25% Avicel RC-581 (FMC BioPolymer) in Eagle’s MEM with 5% FBS was added, and plates were incubated for 48 (INMI-1 isolate) or 72 hours (USA/WA-1 isolate) for virus plaques to develop. After incubation, overlays were removed by aspiration, and the cells were fixed with 10% buffered formalin-containing crystal violet stain for 1 hour. Plaques were counted manually, and PFUs were determined by averaging technical replicates per sample. Percent neutralization was determined relative to IgG isotype antibody control-treated wells.

### Serum PK

Study procedures complied with Animal Welfare Act Regulations (9 CFR 3) and were approved by the Institutional Animal Care and Use Committee of Covance Inc. Serum PK of LY-CoV555 were determined in naïve cynomolgus monkeys, *n* = 3 animals, after administration of LY-CoV555 [5 mg/kg; in 5 mM histidine, 150 mM NaCl, and 0.05% polysorbate 80 (pH 6)] via the intravenous route. At each time point after dosing (predose and 1, 6, 24, 48, 96, 168, 240, 336, 432, 504 576, and 672 hours), 2 ml of whole blood was collected and processed as serum. Samples were analyzed with an immunocapture/MS assay and human IgG enzyme-linked immunosorbent assay (ELISA). Serum PK parameters were determined using a noncompartmental model (Watson, version 7.5 SP1).

### NHP challenge

The rhesus macaque model of SARS-CoV-2 infection was conducted according to the method of Chandrashekar *et al.* (*15*). This study was approved by the Institutional Animal Care and Use Committee of BioQual Inc. in accordance with the animal welfare requirements and accreditations. Housing and handling of the animals were performed in accordance with the standards of the American Association for Accreditation of Laboratory Animal Care International’s reference resource: the eighth edition of the *Guide for the Care and Use of Laboratory Animals*, Animal Welfare Act as amended, and the 2015 reprint of the Public Health Service Policy on Human Care and Use of Laboratory Animals. Handling of samples and animals complied with the *Biosafety in Microbiological and Biomedical Laboratories, 5th edition* (Centers for Disease Control). Naïve female rhesus macaques of Indian origin (purpose bred, *Macaca mulatta* from PrimGen, 8 to 12 years of age) received LY-CoV555 (1, 2.5, 15, or 50 mg/kg) or IgG1 control antibody (50 mg/kg) by slow intravenous bolus (*n* = 3 or 4 animals per group). On study day 0 (1 day after antibody administration), monkeys received a viral challenge of 1.1 × 10^5^ PFU SARS-CoV-2 USA-WA-1/2020 in 2 ml volume administered, divided as 0.5 ml intranasally and 1.0 ml intratracheally. Live phase parameters were monitored before study through necropsy (day 6). COVID-19–specific observations were collected daily in conscious animals to monitor overall health and welfare and determine the need for veterinary intervention or euthanasia. COVID-19 observations were scored on a scale of 0 to 10 and included measures of respiratory rate and dyspnea, overall appearance, activity, and responsiveness. Clinical observations were assessed cage side twice daily and included evaluations of overall animal appearance, fecal consistency, and appetence. Body weights and rectal body temperatures were measured daily in anesthetized animals. Macroscopic observations in the lungs were evaluated at termination on study day 6.

BALF and nasal and oral swabs were collected on days 1, 3, and 6, and lung tissue samples were collected at necropsy on day 6 to assess sgRNA and gRNA via quantitative real-time PCR (qRT-PCR), conducted as previously reported (*15*, *18*). The lower limit of detection for genomic and subgenomic RNA copies was 50. In cases where the values were below the lower limit of detection in the assay, a value of 25 (one-half the limit of quantitation) was used for calculations. This is a common approach for analytical data below the limit of quantification (*75*) and was adopted to provide a conservative estimate. Serum and BALF samples were also assayed for determination of LY-CoV555 concentrations by total human IgG ELISA.

### Immunocapture liquid chromatography–MS assay for LY-CoV555 in cynomolgus monkey serum

The bioanalytical assay for determination of LY-CoV555 in cynomolgus monkey serum is based on a hybrid immunocapture liquid chromatography–tandem MS (LC-MS-MS) method. Briefly, 50 ml of standard, controls, or samples was transferred to a 96-well plate, with 35 ml of SILu MAb K1 internal standard solution (Sigma-Aldrich, catalog no. MSQC6) and 35 ml of biotinylated goat anti-human IgG (100 mg/ml; SouthernBiotech, catalog no. 2049-08), and mixed for 60 min at RT. A 20-ml volume of streptavidin–magnetic beads (Promega V7820) was added to each well, followed by mixing for 30 min. The plate was placed on a magnetic separator, and supernatant was removed, followed by two cycles of washing with PBS. Bound LY-CoV555 was eluted with the addition of 50 ml of 0.1% formic acid, and the supernatant was transferred to a fresh plate, dried down, and reduced with 10 mM tris(2-carboxyethyl)phosphine in 8 M urea for 30 min at 37°C and then alkylated with 10 ml of 50 mM iodoacetamide/50 mM ammonium bicarbonate at 37°C for 15 min. Digestion was performed with the addition of 20 ml of trypsin (10 mg/ml; Promega Cat V511A) and incubated at 37°C for 4 to 13 hours. The reaction was quenched with the addition of 45 ml of 1% formic acid in water. The digested solution was injected onto a Sprite Armor C18 40 × 2.1 mm column (two columns in series) using a Thermo Ultimate 3000 RS LC. Eluant A consisted of 0.1% formic acid in water, and eluant B consisted of 0.1% formic acid in acetonitrile. The 400 ml/min gradient elution profile was initially held at 10% eluant B for 1.5 min, ramped to 50% eluant B at 3.5 min, and then ramped to 80% eluant B at 4 min before returning to 10% eluant B at 4.5 min. The LC column was connected to a Q-Exactive Plus Orbitrap MS using a HESI-II heated ion source. Selective signature peptides from LY-CoV555 and SILu MAb K1 internal standard were detected using targeted selected ion monitoring in the positive ion mode. Mass spectral data were quantitated using QuanBrowser (Thermo Fisher Scientific XCalibur 4.3). Samples analyzed in the LC-MS assay were also analyzed in the human IgG ELISA and demonstrated comparable results.

### ELISA for determination of human IgG concentrations in rhesus macaque serum or BALF

Concentrations of human IgG in rhesus macaque serum or BALF were determined by an ELISA. Goat anti-human kappa monkey ads-UNLB (1.00 μg/ml; SouthernBiotech, catalog no. 2064-01) was coated on the ELISA plate (Thermo Fisher Scientific, catalog no. 3855 or equivalent) as the capture reagent. For the serum assay, calibrators, controls, and samples in neat serum were diluted 200-fold in PBS casein assay buffer and were transferred to the coated plates. For BALF samples, a 10-fold dilution in PBS casein buffer was used. After incubation, the plate was washed to remove unbound material, and mouse anti-human IgG Fc-HRP (horseradish peroxidase) (10 ng/ml; SouthernBiotech, catalog no. 9040-05) was added as detection reagent. After incubation, unbound enzyme was washed away and BioFX 3,3′,5,5′-tetramethylbenzidine one-component HRP microwell substrate (SurModics, catalog no. TMBW-0100-01 or equivalent) was added to the wells. Color development was stopped by the addition of phosphoric acid (Fisher Chemical, catalog no. A260-500 or equivalent), and the optical density was measured at 450 nm with wavelength correction set to 650 nm. Immunoreactivity was determined from calibrators using a four-parameter logistic (Marquardt) regression model with 1/F2 weighting (Watson Bioanalytical LIMS, version 7.4.2 SP1).

### Urea concentration determinations

Urea nitrogen in BALF samples was determined using Abcam 96-well colorimetric urea assay (catalog no. Ab83362) as directed by kit instructions. A 25-μl volume of BALF sample was used for analysis. Standards used in the assay ranged from 1 to 5 nmol per well. The kit directions indicate that the lower limit of detection is 0.5 nmol per well.

Urea nitrogen in serum samples was determined at Charles River Laboratories (CRL)–Mattawan using an automated Beckman Coulter AU5800 chemistry analyzer as directed by the product insert (Beckman Coulter OSR6134, OSR6234, or OSR6634). In this method, urea was hydrolyzed enzymatically by urease to yield ammonia and carbon dioxide. The ammonia and α-oxoglutarate were converted to glutamate in reaction catalyzed by l-glutamate dehydrogenase. At the same time, a molar equivalent of reduced nicotinamide adenine dinucleotide (NADH) was oxidized. Two molecules of NADH were oxidized for each molecule of urea hydrolyzed. The rate of change in absorbance at 340 nm was directly proportional to the blood urea nitrogen concentration in the sample. Serum urea nitrogen was linear from 2 to 130 mg/dl.

### Statistical analysis

In vitro neutralization potencies were estimated using percent neutralization, log10-transformed antibody concentration, and a four-parameter logistic model fit using the drc() package (*76*) with R version 3.6.3 (*77*). All four parameters were estimated from the fitting, and neutralizing concentrations were reported using absolute neutralization concentrations. Overall potency estimates were obtained by meta-analysis of all SARS-CoV-2 neutralization potency estimates using a random-effects model with the metafor R package (*78*).

Because of the left-censored nature of the rhesus macaque viral load data, study sample size, and the need for multiple comparisons correction due to the number of tests being conducted, a multiple imputation approach was favored over a nonparametric testing strategy. Multiple imputation (*m* = 20 imputations) was conducted in accordance with standard procedures described by Rubin (*79*). All statistical analyses were done using log10-transformed viral load values as the response. Imputation of left-censored data was done using random normal values with variance matched to the noncensored viral load values. After imputation, a standard mixed-model repeated-measures model was fit with lme (*80*) using animal as a random effect; group, day, and group × day as fixed effects; and an unstructured covariance matrix. Treatment effects were pooled in accordance with Rubin (*79*) to estimate a pooled effect size, SE, and *P* values. Pooled *P* values were estimated from a *t* distribution, with the degrees of freedom derived from the method described by Barnard and Rubin (*81*). Because of the large number (88) of tests conducted over the combinations of day, dose, sample, and RNA type, *P* values were adjusted for multiplicity using the Benjamini-Hochberg method (*82*). The resultant *q* values from the Benjamini-Hochberg procedure were reported and used to provide control of the false discovery rate.

## SUPPLEMENTARY MATERIALS

stm.sciencemag.org/cgi/content/full/13/593/eabf1906/DC1 Materials and Methods Fig. S1. Recombinant expression, biophysical characterization, and binding kinetics. Fig. S2. Epitope binning heatmap. Fig. S3. Biolayer interferometry measurements of Fab-spike binding kinetics. Fig. S4. Epitope binning communities for 24 selected antibodies. Fig. S5. Structural assessment of antibody epitopes. Fig. S6. Cryo-EM structure validation. Fig. S7. Timeline, screening, and sequence analysis. Table S1. Epitope binding domains and affinities. Table S2. Selected mAb information. Table S3. Hydrogen/deuterium exchange data summary. Table S4. Summary of viral neutralization data. Table S5. Gross pathology findings. Table S6. Statistical analyses for impact of LY-CoV555 on viral loads in SARS-CoV-2–challenged rhesus macaques. Table S7. Serum human IgG concentrations and AUC_0-6days_ after intravenous administration of LY-CoV555 or control IgG to rhesus macaques before and during SARS-CoV-2 challenge. Table S8. BALF and estimated ELF fluid concentrations after intravenous administration of LY-CoV555 or control IgG to rhesus macaques during SARS-CoV-2 challenge. Table S9. Cryo-EM data collection and refinement statistics. Table S10. Crystallographic statistics. Data file S1. SARS-CoV-2 genomic RNA in rhesus model of prophylaxis after administration of LY-CoV555 via the intravenous route. References (*84*–*87*)

## Appendix

View/request a protocol for this paper from *Bio-protocol*.
